# Pathological and Surgical Observations on the Diseases of the Joints

**Published:** 1834-07-01

**Authors:** 


### MEDICAL QUARTERLY REVIEW.

### REVIEWS.

*Pathological and Surgical Observations on the Diseases of the Joints.* By B. C. Brodie, v.p.r.s., Serjeant Surgeon to the King, and Surgeon to St. George's Hospital. *Third Edition.* London, 1834. 8vo. pp. 344.

Science, like history, has its epochs. The eras of history commemorate the foundation or destruction of empires, by the arms of conquerors or the machinations of statesmen: those of science relate only to the establishment of truth and the overthrow of error, through the inspirations of genius or the labours of industry. The former record the progress of mankind from barbarism to civilization; the latter are the registers of the advancement of the human mind from ignorance and imbecility to a state of vigour and intelligence. The historian avails himself of the former as points of sight, from which he may survey and classify past events; they serve, like that "exceeding high mountain," whence were seen the kingdoms of the world, and the glory of them, to enable him to compare their various policy, and their relative degrees of prosperity. The philosopher considers the latter not only as landmarks in his course, but as spots where he may conveniently repose to reflect on his acquisitions, and rejecting, as impediments to his progress, whatever time has shown to be unsound, may dispose all that he has ascertained to be truth so that it shall be useful to him in his subsequent inquiries.

The appearance of Mr. Brodie's papers in the Medico-Chirurgical Transactions formed an epoch in our knowledge of diseases of the joints, and the publication of his third edition, enriched with the experience of twenty years' extensive practice, may with propriety be accounted as another.

As Mr. Brodie's discoveries constitute nearly the Alpha and Omega of our information on this subject, a running commentary on the alterations to be found in the present edition will put us most readily in possession of all substantial improvements; more especially as his candour (always an accompaniment of real merit,) does not fail to appreciate the labours of others, and to award to them that credit to which they are entitled.

Another advantage will accrue from the plan which we propose. There is not a more healthy discipline for the mind than to follow the master-spirits of the world in their researches after truth,—to trace in their company the innumerable windings of that labyrinth in which she is concealed,—to observe with what steadiness they pursue her when once she is discovered, and with what judgment they throw aside the errors with which she is encumbered,—and to witness the struggles by which they draw her from her hiding-place, to exhibit her in all her simplicity and beauty, for the instruction of mankind. Such occupation, in such society, awakens all the powers of the mind, and gives a right tendency to its energies;—instructs it in the signals which indicate the proximity of truth, and renders it familiar with its presence;—accustoms it to oppose the difficulties in its course, and prepares it for setting out single-handed on its own investigations.

There is no science which requires such a discipline more imperatively than surgery, since in it we have no lucky chances or fortunate discoveries, observation and cautious induction being the only methods of prosecuting it with success; and there are no works from which better tuition in these respects may be obtained than from the writings of Mr. Brodie: for, during our comparison of the different editions, we have been struck with his great caution in the admission of any new views, or any modifications of old opinions. We meet with no hasty generalizations, founded on a limited experience, or on a partial consideration of his subject. He revolves each notion in his mind, till convinced not only of its general correctness, but of the accuracy of its minutest details, and then, and not till then, does he bestow on it the approbation of his judgment. Neither do we find in his works any silly remarks about not troubling his readers with cases: in the hands of this powerful alchemist, what others reject as dross is converted into the purest gold. His cases are introduced into every portion of the treatise. Does he wish to describe pathological appearances? a case provides him with the means:—is he about to detail a train of symptoms? it is to a variety of cases that he has recourse. Like the pillars in the temple of Esculapius, they not only record the individual cures which he has performed, but constitute an integral part of that fabric which they at once support and adorn.

But we shall not detain our readers with any more of our remarks, lest they should feel towards us as we have occasionally felt towards the utterers of a long grace before meat; but proceed, without further delay, to set before them the rich entertainment which the author has provided.

The present edition has been greatly enlarged, there being only two chapters that have not undergone some alteration. Many new facts have been introduced, and the descriptions of symptoms have, in some instances, been considerably amplified. The treatment has also received some slight modifications, principally consisting in stricter injunctions as to the preservation of absolute rest, and in the adoption of additional constitutional remedies. Besides these alterations, a new chapter has been inserted on malignant diseases of the joints, and that which treats of various other affections of the articulations has been entirely remodelled. There are, however, no important changes in the pathological arrangements, and we must confess that it was with no small pleasure that we perused the announcement of this fact in the introduction; for, though we are not accustomed, "jurare in verba magistri," yet, such is our respect for Mr. Brodie's judgment, that, had he found occasion to alter his opinions, we should have expected to have had much to unlearn.

The first section, on the *Pathology of Inflammation of the Synovial Membranes*, remains without any alteration till towards the conclusion, where the author is speaking of the occurrence of suppuration in joints without ulceration. The deposition of pus in the different textures of the body has of late been very carefully investigated, and the circumstances under which it is liable to take place are now much better understood than at the period of the publication of the second edition. Mr. Brodie, even at that time, had noticed that this species of suppuration occasionally existed, though he believed that it was only to be found after accidents, or after long-continued or violent inflammation. He is, however, now convinced that such depositions are by no means of unfrequent occurrence, especially in hospital practice; that they are the consequence of a peculiar morbid state of the system generally, following severe accidents or operations, but occasionally met with when the inflammation has not had its origin in any mechanical injury. He enters into no disquisition as to the method in which these deposits are formed, not even so much as to state whether he is a partisan of the theory which supposes them to depend on the absorption of the veins, or of that which attributes them to the agency of the nerves. We may however gather, from his use of the term deposit, that he rejects the simple hypothesis of Dupuytren, viz. that the patient, enfeebled by long illness, is readily affected by exposure to cold, and that the perspiration being suddenly checked, inflammation, of the kind which speedily terminates in suppuration, attacks some of the internal parts. Referring the reader who may be anxious for information on the subject to the papers of the late Mr. Rose and Mr. Arnott, in the Medico-Chirurgical Transactions, the author contents himself with the statement of the facts, and proceeds to detail the following case, in which the secretion of the inflamed synovial membrane was tinged of a dark reddish colour, in the manner mentioned by the latter writer.

"Henry Payne, thirty-nine years of age, was admitted into St. George's Hospital, under the care of Mr. Hawkins, on the 7th of October, 1829.

"He had suffered, formerly, from repeated attacks of rheumatism.

"About twelve weeks ago, after exposure to damp and cold, he was seized with inflammation in nearly all his joints. In the course of a few days, the disease in the other joints had abated; but the right knee became more painful and swollen. At the time of his admission, this knee was tender, painful, and much distended with fluid, and there was a good deal of febrile excitement of the system.

"Blood was taken from the neighbourhood of the knee by cupping; and this was followed by the application of blisters. The vinum colchici, and afterwards calomel, combined with opium, were administered internally. Under this treatment the pain and swelling of the knee subsided.

"On the 27th of October, he was attacked with severe inflammation of the fauces and larynx; which, however, soon yielded to the remedies employed.

"On the 31st, he complained of severe pain in the right side, with great difficulty of breathing; and on the 3d of November he died.

"On examining the body after death, the pleuræ were found inflamed, and incrusted with lymph, and serum had been effused into that of the right side. The lungs also were inflamed, and some portions of them were in a state of gangrene. The heart was affected with hypertrophy, and the pericardium was inflamed with flakes of lymph adhering to it. The synovial membrane of the right knee was full of a dark-coloured fluid; not purulent, but having the appearance of a thick synovia, tinged with blood. The synovial membrane was everywhere of a red colour, as if stained by this secretion, and the cartilages of the joint had the appearance of having been stained in the same manner. There were some small extravasations of blood in the cellular membrane external to the joint." (P. 14.)

In detailing the symptoms of inflammation of the synovial membrane, Mr. Brodie has enlarged upon the characteristics of this affection in the shoulder and hip-joints. The former may be recognized by the swelling of the shoulder at the onset of the disease, and by the crackling, on the motion of the limb, from the effusion of fluid into the neighbouring bursæ. In the latter stages of the malady, the affected joint appears smaller than the other, from the wasting of the deltoid muscle. When this disease attacks the hip-joint, there is some little difficulty in distinguishing it from incipient ulceration of the cartilages. The violence of the pain was only a term of comparison, and in many cases it might be doubtful whether it had not reached the severity required to denote the more serious form of disease. We are, however, now supplied with a diagnostic symptom, which may be readily detected, and which clearly points out the nature of the affection. It is well known that, in the ulceration of the cartilages of the hip-joint, the pain is always referred to the knee; not so in the inflammation of the synovial membrane: the patient, in the latter, always complains of pain in the upper and inner part of the thigh, immediately below the origin of the abductor longus muscle. This symptom, with the aid of others formerly recognized, as pain on the motion or extension of the joint, and the absence of pain on the pressure of the articulating cartilages against one another, will henceforth render the diagnosis comparatively easy.

Another fact mentioned by the author, independent of its intrinsic importance, possesses some interest from its having been known to the surgeons of former days, though of late it has been neglected, and, we believe, its possibility has been denied. Petit, in his "Traité des Maladies des Os," when enumerating the internal causes of dislocation, states, "Il y en a qui sont causées par la sinovie, qui chasse la tête de l'os de sa cavité;" and again, more clearly, "L'amas de la sinovie chasse les os de leur boëte ou cavité; cette liqueur à mesure qu'elle s'accumule éloigne peu à peu la tête de l'os de sa cavité, ce qui est cause de luxation, et souvent d'anchilose."

Sir Astley Cooper has omitted this cause of secondary dislocation, though he has mentioned all the others; and we therefore conclude that he does not believe in its existence.

Mr. Brodie, however, has no doubt that dislocation sometimes arises from this cause; and he not only furnishes us with an explanation of the mode in which it happens, but relates the case of a young gentleman in whom it occurred.

At the conclusion of the section, the author recounts the various modifications of which inflammation of the synovial membrane is susceptible; but, as this summary is replete with original and highly valuable matter, and at the same time is narrated so concisely that it would be difficult to abridge, we shall quote it in his own words.

"It is to be supposed that the character which inflammation of the synovial membrane assumes must, in a great degree, depend on the peculiar constitution of the patient. It is, however, modified by a variety of other circumstances.

"I have already observed, that the symptoms are, for the most part, more severe, and that there is a greater disposition to terminate in the effusion of coagulated lymph, and thickening of the synovial membrane, where the inflammation is strictly local, than where it is the result of some disease affecting the general system.

"In syphilitic cases, it seldom happens that more than one or two joints are affected at the same time. In the early stage of syphilis, the inflammation is usually an accompaniment of a papular eruption or lichen; there is then but little pain; fluid is effused only in small quantity; and when this has become absorbed, the joint is restored as nearly as possible to its original condition. In the more advanced stage of syphilis, we find it existing in combination with nodes: and here it is productive altogether of much more inconvenience to the patient; is more difficult to be relieved; and the synovial membrane is left thickened, and the joint somewhat larger than natural, after the fluid has disappeared. In cases of the last description, it is often impossible to determine, whether the disease can with most reason be attributed to the agency of the syphilitic poison, or to the repeated exhibition of mercury.

"In cases of rheumatism, several joints are frequently affected, either at the same time, or in succession: and the synovial membranes which constitute the bursæ mucosæ and sheaths of the tendons often participate in the disease. There is usually a good deal of pain and swelling, and the joints are often left stiff and enlarged afterwards. Where the inflammation is connected with gout, the pain is generally out of all proportion to the other symptoms of inflammation; and the patient compares his sensations to those which might be supposed to arise if the joint were compressed by a vice, or if it were violently torn open.

"There is a remarkable, yet not uncommon form of the disease, which may be considered as bearing a relation to both gout and rheumatism, but differing from them, nevertheless, in some essential circumstances. The synovial membrane becomes thickened, so as to occasion considerable enlargement of the joints, and stiffness, there being at the same time but little disposition to the effusion of fluid. In the first instance, the disease is often confined to the fingers; afterwards it extends to the knees and wrists; perhaps to nearly all the joints of the body. Throughout its whole course, the patient complains of but little pain; but he suffers, nevertheless, great inconvenience, in consequence of the gradually increasing rigidity of the joints, and the number which are affected in succession. The progress of the disease is usually very slow, and many years may elapse before it reaches what may be regarded as its most advanced stage. Sometimes, after having reached a certain point, it remains stationary, or even some degree of amendment may take place: I do not, however, remember any case in which it could be said that an actual cure had been effected. The individuals who suffer in the way which has been described, are, for the most part, those belonging to the higher classes of society, taking but little exercise, and leading luxurious lives: but there are exceptions to this rule, and the disease occasionally occurs in hospital practice; in men, and even in females, of active and temperate habits." (P. 24.)

The only objection which we can raise to this section on the symptoms of inflammation of the synovial membrane is, that even yet it is not long enough: we would much rather have been supplied with a description of the symptoms of the deposition of pus in the joints from the graphic pen of Mr. Brodie, than have been referred to the papers of Messrs. Rose and Arnott, however highly we may esteem their labours.

We shall now follow our author to the treatment of this disease. In his former editions his principal attention was bestowed on the local treatment, but he has now favoured us with the following directions as to the constitutional remedies to be employed in such cases.

"In cases in which inflammation of the synovial membrane is connected with rheumatism, those remedies may be employed with advantage which are useful in relieving rheumatism in other textures, such as opium combined with ipecacuanha, or other diaphoretics; preparations of the colchicum autumnale, and mercury. Of the two latter, I have found reason to believe that the colchicum is to be preferred, where several joints are affected, and where the synovial membranes which constitute the bursæ mucosæ and sheaths of the tendons participate in the disease. In such cases, the wine of the root of colchicum may be administered in doses varying from fifteen to thirty minims, three times daily, or, in some instances, the acetous extract of colchicum may be given in alterative doses of two or three grains every night. On the other hand, mercury is preferable where only one or two joints are affected at a time, but where there has been a manifest translation of the disease, either from some internal organ, or from one joint to another, the form of mercury most generally useful, under these circumstances, is that of calomel combined with opium; and it should be administered in such doses as to affect the gums, or to produce some other indication of its action on the general system.

"In those cases in which the patient complains of an excruciating grinding pain, or of a sensation as if the joint were torn open, and in which I have already stated that the disease probably bears some relation to gout, the relief produced by the exhibition of colchicum is even more remarkable than in cases of rheumatism; being, in some instances, almost immediate, after leeches and other remedies have been employed to no purpose.

"Where inflammation of the synovial membrane arises from syphilis, it will probably disappear under a well-regulated course of mercury; and where it seems to have arisen from the protracted or injudicious use of mercury, or from mercury acting on a peculiar constitution, sarsaparilla may be given with advantage. This last medicine is especially useful where the affection of the joints occurs in combination with diseases of the bones and periosteum.

"In cases of that peculiar chronic disease, which is described in the concluding part of the last section, in which many joints, and sometimes nearly all the joints of the extremities, are affected in succession, it is of importance that the greatest attention should be paid to the general health, so that it may be maintained in as good a state as possible. As long as he is capable of doing so the patient should take sufficient exercise, daily, to induce a moderate degree of perspiration; he should live on a simple diet, avoiding especially raw fruit and acids, and whatever is not of easy digestion; and taking fermented liquors only in small quantity. The bowels should be kept gently open by means of rhubarb, or compound decoction of aloes, or some other of the same class of aperients. It has appeared to me also that, in these cases, patients have derived benefit from the use of acetous extract of colchicum, exhibited at intervals of six or eight weeks, for ten or twelve successive nights, in small or alterative doses; and still more from the long-continued use of alkalis. The carbonate of potash usually agrees with the stomach better than the pure potash. Ten or fifteen grains may be given twice daily, in the middle of the day and evening, and continued, with occasional brief intermissions, for many months.

"But specific remedies are applicable to only a limited number of cases. For the most part, the disease is to be subdued by being treated as a simple local affection, and in no instance is such kind of treatment to be altogether neglected." (P. 28.)

Our readers will have noticed the frequent use of the word colchicum in the above extract. Mr. Brodie seems, indeed, to place great reliance upon its efficacy, and we have therefore no doubt that he has found it extremely serviceable.

But it must be remembered that of late years much of Mr. Brodie's experience has been amongst the higher ranks of society, of whose order gout is as much the prerogative as champagne and a carriage. It may therefore be doubted whether his practice is not modified in some degree by the change in the constitutions of his patients, and whether, amongst the lower orders, opium, sarsaparilla, and slight tonics, will not be found more serviceable than so debilitating a remedy as colchicum.

In former times, when diseases of the joints were attributed to acrimony of the synovia, it was recommended by Mr. Freke that an incision should be made into the joint, in order to prevent the pernicious consequences of its accumulation. The experience of surgeons soon led them to abandon this fatal practice: it was soon discovered that the admission of air into the cavity of an articulation was speedily followed by caries and abscess.

It has, however, been proposed of late to puncture the knee-joint, when much distended with fluid; and Mr. Brodie, with whom, we believe, the operation, as it is at present practised, originated, thus details the result of his experience as to its safety and advantages; and the circumstances, both in its favour and against it, are so justly stated, that his opinion probably approaches very closely to the truth.

"1st. In a thin person, if a few punctures be made with an instrument a very little broader than a couching-needle, by means of an exhausted cupping-glass applied over the punctures, a large quantity of fluid may be easily abstracted without the smallest danger, and with no inconsiderable relief to the patient. But, while inflammation exists, the relief is not permanent, the fluid being rapidly regenerated; so that in a day or two, or perhaps in a few hours, the swelling is as large as ever. If, on the other hand, the inflammation be already subdued, the absorption of the fluid usually goes on so rapidly, that any more expeditious method of removing it is unnecessary. 2dly. If suppuration has taken place in the joint, (not in consequence of ulceration, but from the surface of the synovial membrane,) a free opening made into it with a lancet will often be attended with the best effects. I have known, under such circumstances, anchylosis to become established almost immediately, and the patient to obtain a speedy cure with an anchylosed joint. The most prudent method of proceeding is to make a puncture with a needle first, and allow a small quantity of fluid to escape, so as to ascertain its nature. If it be not simply turbid serum, but actual pus, the lancet may be used afterwards." (P. 32.)

Mr. Brodie insists at some length upon the importance of rest in this, as in all affections of the joints; not only of that passive rest, which consists in keeping the limb quiet in bed, but of such support as may be derived from appropriate bandages. His description of those which he employs will be found in another part of this review, where we shall enter more fully into the subject of the different means of procuring absolute rest.

Every surgeon must have experienced the annoyance of a stiff joint after the occurrence of inflammation of the synovial membrane; and, if we may judge from the general tone of our author's expressions, and from the ingenuity which he has displayed in his search for a variety of remedies, we should conclude that his opinion of the probability of a perfect recovery is now more unfavourable than formerly. The means which he formerly recommended were frictions with dry powder, or with mercurial ointment and camphor, and the employment of a warm douche bath; to these he has now added the use of blisters and the vapour bath. He has likewise known much "benefit to arise from the use of moxa, in the way recommended by Mr. Boyle: that is, the application of it being so managed, that the heat should penetrate into the soft parts without making an eschar, and scarcely making a blister." In speaking of friction, Mr. Brodie adds this highly useful caution:

"It is, however, a remedy which is applicable only under certain circumstances. We must always bear in mind that friction is useful in relieving some of the effects of disease, but not disease itself; and those who recommend it without attention to this principle, in these and in other cases, will often find it to be productive of very injurious consequences." (P. 38.)

Two additional cases of this affection are introduced; one (to which we formerly alluded,) to exemplify the consecutive dislocation of the hip, and the other to illustrate the good effects of puncturing the knee-joint, and of the exhibition of mercury.

Before we leave this section, we must advert for a moment to one portion of it, which, as it is to be found in the second edition, most of our readers must remember: we mean those cases in which disease of the joint accompanied or followed a purulent discharge from the urethra. We observe that Mr. Brodie always describes this discharge either as "resembling gonorrhœa," or simply "as a purulent discharge;" and in one instance he mentions "that the patient attributed the discharge to the infection of gonorrhœa." We are not certain whether these expressions have been employed accidentally, or whether they indicate the author's doubts as to the occurrence of inflammation of the synovial membranes as a consequence of gonorrhœa. We can scarcely believe the latter, since, in our own experience, (limited indeed, when compared with Mr. Brodie's,) several cases in which this has distinctly taken place have come under our observation. In one instance, the gentleman had been under our care for three weeks with a gonorrhœa, of the nature of which there could be no doubt, when he was attacked with inflammation of the synovial membrane of the right knee, which yielded to the treatment recommended by the author, viz. rest, the application of leeches, and the use of colchicum.

The chapters on *Ulceration of the Synovial Membrane*, and of *the Morbid Change of its Structure*, remain as in the former edition; we shall therefore pass over them, to the *Pathological Observations on Ulceration of the Articular Cartilages.* In the following sentences, the various causes of this disease are concisely stated.

"1st. It may be the consequence of disease originating in the neighbouring soft parts, especially of inflammation of the synovial membrane; examples of which will be found among the cases related in the preceding chapters.

"2dly. It may depend on a morbid condition of the cartilage itself; or,

"3dly. On a chronic inflammation of the surface or substance of the bone with which it is connected.

"4thly. It may be the result of a peculiar alteration in the condition of the cancellous structure of the bones, which is met with in young scrofulous persons." (P. 95.)

In the former edition no mention was made of ulceration of the cartilage occurring as a secondary disease, the primary disease having had the character of rheumatic inflammation of the periosteum and bone. The following case so well exemplifies its progress, and the morbid appearances after death, that we quote it at full length.

"Sarah Holder, twenty-two years of age, was admitted into St. George's Hospital, on the 26th of July, 1827, with a diffused swelling extending from the upper part of the right thigh to the leg, a little below the knee. The swelling was most conspicuous in the immediate neighbourhood of the knee-joint; and from thence gradually became diminished, having no defined termination either above or below. It was somewhat elastic, the skin over it appearing glossy and tense, but not redder than natural. The patient complained of exquisite pain, especially on pressure. The pain was also aggravated by every motion of the knee, nevertheless it was principally referred, not to the joint itself, but to the thigh-bone immediately above it. In addition to these local symptoms, the pulse was frequent; the tongue furred, and rather brown; the skin hot; and the countenance anxious, and expressive of much suffering. The condition of the patient was altogether a good deal similar to that which might be produced by severe rheumatic inflammation of the bone and peritoneum; and the history of the case seemed to justify the opinion that such was the nature of the disease, as the symptoms had begun without any precursory rigor on the day previous to her admission, and had been preceded, for an entire month, by rheumatic pains in the elbows and shoulders.

"Saline and antimonial medicines were exhibited: leeches were freely applied to the limb, and on the 28th of July, a pill, containing two grains of calomel and half a grain of opium, was exhibited twice daily. Under this treatment the gums became slightly affected, and the symptoms gradually abated. On the 3d of August, the mercurial pill was given only once daily; and, in the course of a few days more, it was altogether discontinued, blisters being at the same time applied to the limb.

"August 13. The swelling and pain had entirely left the upper part of the thigh; but there were still some remains of both in the immediate neighbourhood of the knee. Altogether, she was in a much better state with respect to the local symptoms, and the general health was improving.

"August 15. After an accidental exposure to cold, she had a rigor, followed by fever; and, at the same time, there was a recurrence of pain and swelling in the neighbourhood of the right knee, with some degree of pain and tenderness extending up the thigh, and down the leg. The swelling had the same character as formerly.

"August 20. She continued in nearly the same state, with painful startings of the limb, and perspirations at night. Pulse very frequent. She was directed to resume the use of calomel and opium.

"September 2. There was no material improvement as to the local symptoms: a blister was applied to the knee.

"She continued in nearly the same state, sometimes a little better, sometimes a little worse, with a very frequent pulse, and the general health, on the whole, declining, until the 7th of October; when an issue was made with caustic in the neighbourhoood of the knee. The issue seemed to occasion some abatement of the local symptoms. Her bodily powers, however, continued to decline, and she became affected with an ulcer over the sacrum, the result of long-continued pressure.

"October 14. She complained of severe pain in the left shoulder.

"October 15. She was seized with a vomiting and purging, accompanied with pain and tenderness of the abdomen and cold extremities. Pulse 140. At midnight she had a severe rigor.

"The vomiting and purging continued, in spite of the remedies which were employed. In the afternoon of October 16, she had another rigor, and in about two hours afterwards she expired.

"On examining the body, the knee-joint was found to contain neither pus nor synovia. The cartilage of all the bones which enter into the composition of the joint were ulcerated in several places, especially that of the inner condyle of the femur. A slight extravasation of blood had taken place into the cavity of the joint, apparently from the surfaces of the bone exposed in consequence of the ulceration of the cartilages. The periosteum could be easily peeled off the surface of the femur, and the bone underneath appeared to be more vascular than is natural. The stomach was distended with an acid fluid of a green colour, similar to what had been vomited on the day preceding death. The gall-bladder was full of a very pale yellow fluid. There were no other morbid appearances.

"The left shoulder, to which pain had been referred for a short time previous to death, was carefully examined, but no disease was detected in it." (P. 119.)

A case of the same nature is related in a subsequent part of the work, which terminated successfully, under the use of calomel, and the employment of a caustic issue.

In the history of the symptoms of ulceration of the cartilages, there are only a few verbal alterations, which we must omit, in order to bestow more attention upon the treatment of these affections. The only additions to the constitutional remedies are a full course of sarsaparilla, in such cases as are connected with a chronic inflammation of the bone in the neighbourhood, and the employment of mercury in that secondary form of the disease to which we have before adverted. Mr. Brodie is still in the habit of employing caustic issues, and other counter-irritants, locally, and speaks in the same decided manner of their utility in this form of the disease. But the great sheet-anchor to which he attaches the most importance, is rest. His directions on this subject are copious, but so valuable to the practical surgeon, that we shall quote them without abridgment. We shall at the same time introduce his account of the various bandages which he habitually employs for preventing the motion of the joints affected with inflammation of the synovial membrane.

"When the cartilages of the hip are ulcerated, the patient should be confined to his bed or couch, being never allowed to move from it on any occasion. If left to himself, he is generally inclined to lie on the side opposite to that of the disease. There are, however, good reasons why this position should be avoided, if possible. It necessarily distorts the pelvis, and increases the disposition to a lateral curvature of the spine. It also, in those cases in which the round ligament of the joints is destroyed, facilitates the escape of the head of the femur from the acetabulum, and the production of dislocation. Something may be done towards preventing this last effect, by interposing a pillow, or thick cushion, between the knees; and it is difficult to do more than this, after the patient has already been lying on his side for a considerable time: otherwise he should be placed on one of the bedsteads invented by Mr. Earle, lying on his back, with the shoulders and thighs somewhat elevated, and the latter as nearly as possible parallel to each other. This supersedes the necessity of having recourse to splints and bandages, and, with a view to the confinement of the hip-joint, is all that is required in the early stage of the disease.* At a later period, when, in consequence of the extensive destruction of the articulation, the muscles begin to cause a shortening or retraction of the limb, I have found great advantage to arise from the constant application of a moderate extending force, operating in such a manner as to counteract the action of the muscles. For this purpose an upright piece of wood may be fixed to the foot of the bedstead, opposite the diseased limb, having a pulley at the upper part. A bandage may be placed round the thigh above the condyle, with a cord attached to it, passing over the pulley, and supporting a small weight at its other extremity. I will not say that the effect of such a continuance is to prevent the shortening of the limb altogether; but I am satisfied that it will, in a number of instances, render it less than it would have been otherwise, at the same time preventing, or very much diminishing that excessive aggravation of the patient's sufferings with which the shortening of the limb is usually accompanied." (P. 145.)

"Of the above observations on the ulceration of the cartilages of the hip, many are applicable to the disease in other joints.

"In all cases it is indispensable that the parts affected should be kept in a state of the most complete repose, and this is to be accomplished by various means, accordingly as one or another joint is the seat of the disease. In some instances, when the disease is in the knee, or ankle, or tarsal joints, nothing can be done better in the first instance than simply to lay the joint on an air-pillow, which, if not much distended with air, gives an uniform, regular, and most convenient support on every side; but, for the most part, it is better to have recourse to splints made of pasteboard, or stiff leather, neatly moulded to the figure of the limb. When the disease is in the shoulder, the forearm should be supported by a light leathern coat, suspended from the waist or neck, and the arm should be kept constantly bound to the side; and when it is in the ankle, great advantage will often arise from the patient wearing a common wooden leg, which will enable him to

* "On some occasions, however, it is convenient to fix the pelvis by a strap or bandage, passing over it, from one side of the bedstead to the other; and even the thigh may be fixed in the same manner."

take exercise for the maintenance of his general health, without aggravating the local disease." (P. 153.)

"If the disease be far advanced, and there is danger of the cartilages being ulcerated, he will find it prudent to restrain the motions of the joint altogether, by the application of pasteboard splints, confined by a roller, or even by circular stripes of adhesive plaster on their outside. In other cases, the bandages, &c., recommended by Mr. Scott, in his ingenious work on the diseases of the joints, will be productive of the best results.* There is a bandage which is very well suited to cases of this kind, which, in one part of its circumference, is composed of a stiff leather, elsewhere of an elastic material, and secured by a lace or buckles, so that it admits of being secured with any degree of tightness. If the seat of the disease be in the knee, there may be a single piece of leather, adapted to the shape of the posterior part of the limb; if it be in the elbow, there may be a double piece of leather, one on each side, and thus the construction of it may be varied so as to adapt it to any of the other articulations. In some instances much support may be wanted, and the leather should be stiff and unyielding; extending a considerable way above and below the joint. In others, where little support is necessary, the leather may be more pliant, and it should not extend beyond the immediate neighbourhood of the part affected." (P. 36.)

In the above passage Mr. Brodie alludes to Mr. Scott's method of bandaging; and, as all of our readers must have heard of that gentleman's success, though they may not be acquainted with the means which he employs, we shall quote his account of them, from his work on Diseases of the Joints.

"In the first place, the surface of the joint, suppose the knee, is to be carefully cleansed by a sponge, soft brown soap, and warm water, and then thoroughly dried; next, this surface is to be rubbed by a sponge soaked in camphorated spirit of wine, and this is continued a minute or two until it begins to feel warm, smarts somewhat, and looks red. It is now covered with a soft cerate, made with equal parts of Ceratum Saponis and the Unguent. Hydr. fort. c. Camph. This is thickly spread on large square pieces of lint, and applied entirely around the joint, extending for at least six inches above and below the point at which the condyles of the femur are opposed to the head of the tibia: over this,

* "A very convenient mode of applying bandages in these cases is as follows: Let it be supposed that the disease is in the knee. Circular stripes of leather, spread with the Emplastrum Plumbi, are to be applied round the joint, and extending some way above and below it; care being taken that a space is left for the patella, on which there ought to be as little pressure as possible. Over this a calico roller (four or five yards for an adult,) may be applied, and over this again a few circular stripes of linen, spread with adhesive plaster, with another calico roller over the whole. A bandage of this kind, carefully adjusted, may not require to be changed for six or eight weeks, and is very convenient to the patient."

to the same extent, the limb is to be uniformly supported by stripes of calico spread with the Empl. Plumbi of the London Pharmacopœia. These strips are above one inch and a half broad, and vary in length: some are fifteen inches, others a foot, others half these two lengths, and the shorter or longer are selected according to the size of the part round which they are to be applied. This is the only difficult part of the process. This adhesive bandage ought to be so applied as to preclude the motion of the joint, prevent the feeble coats of the blood-vessels being distended by the gravitation of their contents in the erect posture, and thereby promote their contraction. Over this adhesive bandage, thus applied, comes an additional covering of Empl. Saponis, spread on thick leather, and cut into four broad pieces; one for the front, the other for the back, the two others for the sides of the joint. Lastly, the whole is secured by means of a calico bandage, which is put on very gently, and rather for the purpose of securing the plaster, and giving greater thickness and security to the whole, than for the purpose of compressing the joint. This is an important point, as otherwise an application, which almost invariably affords security and ease, may occasion pain, with all its attendant mischief." (*Scott on Diseases of the Joints*, pp. 133—135.)

We observe, in page 142 of Mr. Brodie's book, that he attributes the utility of Mr. Scott's plan of treatment to its maintaining the diseased joint in a state of absolute immobility; and we have no doubt that, if it did not do this, it would be utterly useless: but, on the other hand, we agree with Mr. Scott, that the constant excitation of the cutaneous vessels, and their exposure in this state to a powerful mercurial preparation, cannot be without its advantages. It must contribute to the absorption of the lymph effused in the neighbourhood of the diseased joint, and thereby put the vessels of the part into a more healthy condition; while, at the same time, it acts as a counter-irritant, in checking any chronic inflammation that may be going on within.

The section on *the Pathology and Symptoms of Scrofulous Disease of the Joints, arising in the Cancellous Structure of the Bone*, has been improved by the insertion of an additional case, and by an excellent description of the peculiar form of the swelling characteristic of this affection of the superficial joints. In speaking of the use of counter-irritants in this disease, Mr. Brodie states that he has known them of some service in certain instances of disease of the hip, where there was an unusual degree of pain and muscular spasm, attributable to irritation of the anterior crural nerve; but he continues to think that, in the great mass of these cases, they are certainly of no service, if not absolutely prejudicial. In the treatment of the constitution, he relies but little upon iodine, much as it has been lauded by some surgeons as a specific for scrofulous disease. His experience is, that it is useful in a few cases, and in very small doses; but he believes that the large doses in which it is sometimes exhibited have a tendency to diminish, rather than to increase the vital powers. He strongly recommends the use of liquor potassæ in full doses, combined with light bitters, and continued for a very long period. Great care should be taken to regulate the action of the bowels, and none but the plainest and most nutritious food should be permitted.

The author enters at considerable length into the question of the amputation of scrofulous joints, and his opinions are somewhat modified by his late experience. In his last edition he dwelt much upon the liability of the patient to visceral diseases, and his conclusions were summed up in these words:

"I do not say that these considerations are sufficient to warrant the surgeon in forbidding an operation altogether, in all cases where it is not actually and indisputably necessary to save the patient's life; but they are certainly sufficient to make him cautious not to recommend and urge it too strongly. They show the prudence of delay in certain cases. Perhaps, after the lapse of one, or two, or more years, by means of proper medicines and a judicious attention to diet and mode of life, and still more in consequence of that change which the mere lapse of time may produce in the constitution of a young person, the patient's general health may be so far improved, that the diseased joint may be removed without that risk of subsequent mischief which would have been incurred at a former period." (Pp. 268-9, *second edition.*)

He has now adopted a rather more favourable opinion of these cases: he believes that the removal of the limb may, in some instances, prevent or suspend the visceral disease. But, in order to enable our readers to judge how far Mr. Brodie has modified his opinions, and upon what good grounds he has done so, we shall gratify them by quoting the passage.

"But we may refer to another order of facts, as shewing that there are occasions in which the amputation of a scrofulous joint, instead of rendering other organs more liable to the same disease, may actually produce the opposite effect of preserving them from it. It is to be observed that such a disease of a joint is never more than the remote cause of death, and that, where the result is fatal, it invariably happens in the following manner. The patient is exhausted by hectic fever, and in this state of debility disease takes place in the mesentery or lungs, or not unfrequently in both these parts at the same time, and it is this visceral affection which immediately precedes dissolution. It is evident, then, that in many cases there is a period of time at which the amputation of the limb may be the means of preventing the establishment of a secondary disease. Nor is this all. Visceral disease, which was previously in a state of inactivity, may assume a new form, and begin to make a rapid progress, under the influence of the disease of the joint; and amputation, under these circumstances, may be the means of preserving the patient, if not altogether, at least for a considerable time, perhaps for several years. A young woman was admitted into the hospital labouring under a scrofulous affection of the ankle. It was of long standing, and there were several abscesses communicating with extensive surfaces of carious bone. It was evident that there was no chance of cure for the disease in the joint. Nevertheless, I did not think it right to propose to the patient that she should submit to the loss of the limb, as she had a troublesome cough, with a purulent expectoration, and other marks of pulmonary disease. She however earnestly implored that the ankle might be removed, and at her request, and certainly against my own judgment, I performed the operation. The stump healed readily. The pulmonary symptoms almost immediately subsided. She lived for four or five years in tolerable health, but at the end of that period (as I have been informed,*) there were again manifest indications of disease within the chest, of which she ultimately died.

"It is evident, from these statements, that the question concerning amputation, is, in many instances, one of a complicated nature, requiring the exercise of no small degree of judgment and discrimination on the part of the surgeon, and not to be determined, except after a minute investigation of the whole case, with respect to the disease in the joint itself, and also in whatever relates to the state of the general health at the time, and that of the constitution previously." (P. 210.)

The subject of *Anchylosis* has also occupied Mr. Brodie's attention. His opinions are briefly these: that ulceration of the cartilages may exist without anchylosis being the consequence, but that in bad cases adhesions are formed in proportion to the severity of the attack. He gives the following excellent directions as to the conduct of the surgeon during its formation.

"It is never prudent to have recourse to any mechanical means for the purpose of preventing anchylosis taking place, lest a fresh attack of inflammation and abscess should be the consequence. We may however venture, when the circumstances of the case require it, to adopt measures for the purpose of gradually placing the limb in a more commodious position. For example: when the knee has been affected, if left to itself, it often happens that the leg becomes fixed at a right, or even an acute angle with the thigh; and a light apparatus may be applied to the limb, with a screw at

* Mr. Brodie has been misinformed as to the termination of this case. We have had an opportunity of seeing the patient of late, and she is perfectly healthy, and without any marks of visceral disease.

the posterior part, by the agency of which the leg may be very slowly and cautiously extended. In like manner, if the elbow be in danger of being anchylosed in the straight position, it may be very gradually brought into a state of flexion. It is scarcely necessary to explain wherefore, in the knee-joint, the straight position is to be preferred to the bent; while in the elbow it is desirable to obtain the latter position instead of the former." (P. 213.)

Two additional cases are introduced; one to illustrate the pathology of the disease, and the other to indicate the treatment which the author has of late found most efficacious.

If we were called upon to point out the portion of the work which has undergone the greatest improvement, we should certainly select that upon *Caries of the Spine.* The chapter on its pathology has been entirely remodelled. The various origins of the disease are more clearly arranged, and the whole exhibits that peculiar process of simplification with which it appears Mr. Brodie's object (as it should be that of every scientific man,) to invest every subject that he handles. There are, however, no new facts which especially require our notice; and, as we have already been rather lengthy, and have still much before us, we must proceed, without making any comments upon this part of the subject.

The symptoms of caries of the spine are of two orders; one connected with the disease of the bone, the other the effects of the disturbance of the nerves or spinal marrow. Mr. Brodie thus briefly enumerates them:

"1st. Pain and tenderness in the situation of the carious vertebræ.

"2dly. Curvature of the spine forward, with an angular projection of the spinous processes posteriorly, the result of the bodies of the vertebræ having been destroyed, while the other parts of these bones remain entire.

"3dly. Abscess commencing imperceptibly, but at last presenting itself as an external tumor.

"4thly. Pains, loss of sensation, coldness, muscular spasms, and paralysis of the extremities.

"5thly. Derangement of the functions of the various viscera, which are capable of being influenced by that portion of the spinal chord which is implicated in the disease." (P. 249.)

Of these it is of the most importance to study well the nature of the pain, as it is the only symptom which indicates the onset of the disease. The following is Mr. Brodie's description of it.

"In the majority of cases, the first symptom which the patient notices, is pain referred to that part of the spine in which the caries exists; at first trifling, but becoming more severe afterwards. The pain is aggravated by any sudden motion of the spine; by percussion, or by a jar communicated to it in any other way; as by stamping on the ground, striking the foot accidentally against a stone, sneezing, or coughing. In the advanced stage of the disease the pain is sometimes so severe, and so easily induced, that the patient cannot bear the slightest movement. Yet, in other cases, there is sometimes no pain in the spine whatever, from the first access of the disease to its termination." (P. 249.)

Then follow two cases in which the patients died from the disease, without once complaining of the slightest pain in the affected part.

Concerning the *Curvature*, the author only adds one remark, viz. that it can never exist until the disease has made some progress, during a period which varies from three months to two years.

The same observation applies to the formation of *Abscess*, with this addition, that, after it is formed, it may lie dormant for a long period, without affording any indication of its existence. It is however "usually attended with some derangement of the general health, such as loss of flesh and muscular power; increased frequency of the pulse; a slight access of fever in the evening, followed by perspiration at night; occasionally, but rarely rigors." But every surgeon knows that the symptoms vary according to the portion of the spine that is affected; and it is in his detail of these symptoms that the author has made such valuable additions to this part of his work. We can however afford room only for a small portion of them, and we shall therefore select, as a specimen of the whole, his description of caries of the cervical vertebræ.

"When there is caries of the cervical vertebræ, the patient complains, in the first instance, of pain in the neck, which is aggravated by every motion of the head, and which is not unfrequently mistaken for the muscular pains and stiffness connected with what is commonly called a stiff neck from cold. The pain gradually increases, and, according to my experience, is more liable to be severe than when the seat of the disease is in the lower part of the spine. When, in the progress of the disease, the spine has become incurvated forward, the angular projection posteriorly is observed to be trifling, except when the lowest or seventh cervical vertebra is implicated in the disease; a difference which is easily explained by the greater length of the spinous process of the latter, as compared with that of the spinous processes of the vertebræ above.

"Abscess connected with diseased cervical vertebræ usually presents itself among the muscles on the side of the neck. Occasionally it makes its way forward, forming a tumor, and afterwards breaking in the pharynx. I have seen one case in which the abscess penetrated into the theca vertebralis, and the whole of the spinal chord, from its origin to its termination, was bathed in pus. At an early period of the disease, the patient frequently complains of pains in the arms and shoulders. After some time these pains subside, but they are followed by complete paralysis of the upper extremities; while the muscles which derive their nervous influence from the spinal chord below the neck remain subject to the will. In a still more advanced stage of the disease, the paralysis extends to the muscles of the trunk and of the lower extremities. Then there are pains in the abdomen, which becomes distended and tympanitic, the bowels being at the same time obstinately costive. In all cases, there is pain in the occiput and temples; which is, however, most severe when the disease is situated in the two or three superior vertebræ. Not unfrequently the transverse ligament of the second vertebra is destroyed, and the consequence is, a dislocation of the odontoid process. Sometimes the dislocation is complete, and the patient, from the pressure made on the spinal chord, expires as suddenly as if the latter had been divided transversely. More frequently it happens that the displacement of the odontoid process is somewhat restrained by the pressure of the dura mater which lies over it. There is then some degree of pressure on the spinal chord, sufficient to excite irritation, but not sufficient to destroy its functions. Under these circumstances, the patient complains of increased pain in the head, followed by convulsions, stupor, dilated pupils, and other symptoms of effusion of fluid on the brain; and on examining the body, after death, we find that such effusion has actually taken place, there being a collection of fluid in the ventricles, or in the base of the cranium, or in both of these situations." (P. 254.)

We greatly regret that we are obliged to omit the author's description of *Disease of the Dorsal and Lumbar Vertebræ*, with an excellent account of what is commonly called *Psoas Abscess.* It is seldom that such exquisite morsels fall in the way of a reviewer, and it is therefore with great difficulty that we turn our back upon them, to proceed to the directions concerning the treatment of these cases. This may be briefly stated to consist in the employment of rest, (and the author prefers the use of Earle's bedstead,) and of issues in the neighbourhood of the diseased part. Mr. Brodie has long known that the latter were useful in some cases, and nearly useless in others, and his knowledge of their effects on diseases of the joints had led him to suspect that they were useful only in those cases where the disease did not commence in the cancellous structure. His subsequent experience has confirmed this opinion, and his present language is as follows:

"Nor, if my observations on the subject be well founded, is this to be regarded as a merely theoretical opinion. I have repeatedly known the greatest relief to follow the establishment of issues, where the patient has suffered severe pain in the situation of the carious vertebræ, presenting at the same time no distinct indications of a scrofulous diathesis; while in young persons, with fair complexions and dilated pupils, in whom the disease has proceeded with little or no pain, they have appeared to be either inefficacious, or actually injurious. It appears to me also that in caries of the spine, as well as in that of other joints, issues are to be employed only in the early stage of the disease, with a view to prevent suppuration, and that they are of no service after abscess has actually formed." (P. 268.)

We pass over the chapter upon *Tumors and Loose Cartilages in the Cavities of the Articulations*, as it remains unaltered, and we now approach the new chapter on *Malignant Diseases of the Joints*. These are of two kinds, carcinoma, and fungus hæmatodes. First, as respects carcinoma. Mr. Brodie has never known this to exist as a primary disease in the bones, but it has always been preceded by a similar affection of the breast, or some other glandular organ. Its existence has been indicated by pseudo-rheumatic pains, of different degrees of severity, and by extreme brittleness of the bones. Two cases are detailed; one of disease in the hip, which may be found in the second edition, Case LX., and the other of disease in the vertebræ. We quote the latter for the instruction of our readers.

"CASE LXXI. A lady, about thirty-eight years of age, consulted me, in the spring of 1832, on account of a scirrhous disease of one breast. There was not a distinct scirrhous tumor imbedded in the substance of the breast, but a conversion of the gland itself into the scirrhous structure. The skin covering the breast was thickened, and manifestly contaminated by the disease.

"From this time I saw her occasionally, the disease in the breast making little or no apparent progress.

"During the night of the 10th of February, 1833, she suddenly became paralytic in the whole of the lower part of her person. She not only lost the power of using her lower limbs, but that of voiding her urine also; and it became necessary to empty the bladder by means of a catheter.

"The loss of muscular power was attended with a loss of sensibility as high as the navel and lowest dorsal vertebræ. When the catheter was introduced into the bladder, she was not sensible of its introduction.

"In the beginning of March the lower limbs became affected with involuntary convulsive movements, which were unattended by pain, but of which the patient complained that it was disagreeable for her to see them.

"When the paralysis first took place the urine was clear, and otherwise in a natural state; afterwards it became ammoniacal and offensive to the smell, depositing a thick mucus, with traces of phosphate of lime in it.

"On the 9th of April, 1833, the patient died.

"The body was examined by Mr. Cutler, who found the whole of the gland of the breast to have assumed a scirrhous structure.

"Several of the dorsal vertebræ were converted into a substance possessing considerable vascularity, of a gristly consistence, some of them containing no earthy matter whatever, so that they could be cut with a knife. Altogether the alteration in the condition of the vertebræ seemed to be very similar to that which had taken place in the head of the femur, in the case which was last described, except that, being more complete, it might be supposed to indicate a more advanced stage of the disease.

"The whole of the lower portion of the theca vertebralis was filled with a serous fluid.

"There was a deposit of earthy matter in the upper part of each lung; and about four ounces of serous fluid were contained in the cavity of the right pleura.

"The kidneys were of a dark colour, and highly vascular.

"The mucous membrane of the bladder bore marks of considerable inflammation. The ureters, pelves, and infundibula of the kidneys, were also inflamed, and in some parts lined with coagulated lymph. They were considerably dilated, and contained a putrid mixture of urine and mucus." (P. 283.)

Fungus hæmatodes more frequently attacks the bones than carcinoma, and, unlike it, occurs there as a primary disease. It is characterized by a slight degree of pain in the affected part, which is aggravated by exercise. The bone gradually enlarges, presenting a tumor, elastic in some parts, and hard in others, till, after some time, the motions of the joint are totally impeded. It is almost unnecessary to say that amputation, which is the only remedy, frequently fails in removing the disease from the system. We have abridged the following case, to illustrate the progress of this disease in the knee.

"Mr. O., twenty-five years of age, in January, 1828, first experienced a sensation of weakness in the right knee, with a slight pain, after walking even a short distance. These symptoms continued; and, in the course of two or three months, he observed a small tumor over the external condyle. * * *

"On the 25th January, 1829, he came to London. * * At this time there was a very considerable enlargement of the whole of the upper part of the knee-joint, so that it was four inches in circumference larger than the corresponding part of the opposite limb. The tumor was soft and elastic, occupying the situations of both condyles of the femur, but being more especially prominent in that of the outer condyle. The head of the tibia and the patella did not seem to be implicated in the disease, and the joint retained nearly its natural degree of mobility. * * *

The limb was amputated on the 6th of July, 1829.

"On examining the amputated limb, the femur was found to terminate abruptly about five inches above the knee-joint. In place of the condyles and lower part of the shaft of that bone, there was a large tumor, of an irregular form, of that structure to which the name of fungus hæmatodes is commonly applied. The cartilage which had covered the surface of the condyles of the femur was seen expanded over the lower part of the tumor, being everywhere thinner than natural, but nowhere in a state of ulceration. In some parts it had contracted adhesions to the cartilage covering the head of the tibia.

"In other parts the tumor was covered by some thin remains of the periosteum, and a layer of thickened cellular membrane." (P. 286.)

The chapter *on some other Diseases of the Joints* has, as we have before said, undergone great alteration. In the former edition, the first two of these which were mentioned were abscess and exfoliation of the articulating extremities of the bones: in the present they are placed on a much more scientific footing, being treated of as acute and chronic inflammation of the epiphyses, the acute terminating in exfoliation of the bone, and the complete destruction of the joint; the chronic producing enlargement of the epiphysis, and, in more severe cases, occasioning the formation of abscess in the centre of the bone. In the former there is no remedy but amputation; in the latter, where there is only enlargement, sarsaparilla, mercury, iodine, and mezereon, with the application of blisters, will be found useful. When abscess in the bone takes place the pain is intensely severe, and formerly such cases were condemned to amputation; but Mr. Brodie has revived an operation of Mr. Hey's, and employed it with success in several instances. He trephines the bone, opens the abscess, and gives exit to the matter. The difference between Mr. Hey's and Mr. Brodie's operation is this, that Mr. Hey only applied it to such abscesses as had already burst, and found an external opening. Mr. Brodie has recognized the symptoms of this disease at its commencement, and, by practising the operation at an early period, has saved his patients many months (perhaps years) of intense suffering. The particulars of the operation, and several cases in which it was performed, may be found in a paper, by our author, in the seventeenth volume of the Medico-Chirurgical Transactions.

Mr. Brodie has enlarged upon the treatment of hysterical affections of the joints, and he recommends the usual routine of remedies which our empirical practice in this species of disease has for a long time, though very unsatisfactorily, led us to adopt. The following directions are not sufficiently attended to in general, and we therefore avail ourselves of the opportunity of quoting Mr. Brodie's high authority on these points. After recommending various medicines, he says,

"But none of these remedies will do for the patient what may be accomplished by other means. Her attention should be as much as possible withdrawn from the subject of her complaints, and directed to other objects. She should be encouraged to take exercise out of doors, especially on horseback; to rise early, so that only a moderate number of hours may be passed in bed; to live in a cheerful society, and if she has abandoned them (which has too frequently happened,) to resume, in all respects, the habits of a healthy person." (P. 304.)

Mr. Brodie notices another circumstance in connexion with the diagnosis of the disease of the hip-joint which is not generally known, viz. that, independently of disease, the two lower extremities are not always of the same length. The following passage, containing his experience on this subject, must conclude our quotations, and our notice of his work.

"It occasionally happens that the two lower extremities are not of precisely the same length; and this may be the result of original formation, the femur and tibia of one side being respectively longer than those of the other side. If the whole of this difference amounts, as it sometimes does, to an inch, or an inch and a half, the individual is observed to limp in walking, and the great trochanter belonging to the longer limb is higher and more prominent than that of the other; and this might lead a superficial observer to mistake the case for one of diseased hip.

"In some instances there is a difference in the length of the two lower limbs, in consequence of disease. A diseased bone for the most part does not keep pace in its growth with the other parts of the body; but I have known the reverse of this to happen, of which the following is a remarkable instance:

"Case lxxix. Master M. was brought to me for my opinion, in June, 1832. I saw him in consultation with Dr. Lefevre, physician to the British Embassy at St. Petersburgh.

"The cicatrices of three or four abscesses were seen in the skin on the anterior and upper part of the thigh, and there was considerable thickening of the deep seated soft parts in the same situation, there being also a manifest adhesion of them to the bone. The appearance of the limb was such as would lead to the belief that there was a portion of diseased or dead bone of the femur, with probably some new bone formed round it; and that this had produced a succession of abscesses of the soft parts, as in ordinary cases of necrosis. The history of the case seemed to justify this opinion as to the nature of the disease.

"Three years and a half ago the little boy had been suddenly seized with severe pain, which was referred to the knee, but only for a few hours, at the end of which time it shifted its place to the upper and anterior part of the thigh. The pain continued, and swelling immediately took place. At the end of six months an abscess was opened, which however soon healed. Afterwards a second abscess formed, which was followed by others; but all of them had healed without any exfoliation having hitherto taken place.

"There was some degree of stiffness of the hip-joint, but no more than might be reasonably attributed to the thickening and swelling of the soft parts in the neighbourhood. But the most remarkable circumstance in the case was, that the diseased thigh-bone, when measured from the anterior spinous process of the ilium to the patella, was found to be at least an inch and a quarter longer than that of the sound limb. The measurement was made repeatedly and with the greatest care, so that there could be no mistake respecting it. There was no perceptible difference in the length of the bones of the two legs.

"In consequence of one limb being thus longer than the other, when the patient stood erect, with the soles of his feet planted on the ground, the great trochanter on the side of the disease appeared to project unnaturally, and this occasioned a manifest alteration in the form of the nates, somewhat corresponding to what is observed in the less advanced stage of disease of the hip-joint. That this appearance of the nates was to be attributed solely to the difference in the length of the two limbs was proved by this circumstance, that it was at once removed by placing a book an inch and a quarter in thickness under the foot of the sound limb, so as to raise that side of the pelvis to the same level with the other." (P. 212.)

Our readers may now form some opinion of the excellence of the late additions to Mr. Brodie's book: they may perceive that they are characterized by the same accuracy of observation, and fidelity of description, which distinguish all his writings. But, in endeavouring to estimate the merits of the whole volume in its present complete form, it should be borne in mind, that there are two orders of works in medical science of equal value. The one establishes general principles as a foundation for future investigation; the other raises the superstructure by the accumulation of details; the former would be useless without the latter, and the latter unsafe without the former:

"alterius sic

Altera poscit opem res, et conjurat amicè."

The former may claim the palm for the brilliancy and grandeur of its discoveries; but the latter has an undoubted precedence in practical utility. The names of Harvey, of Hunter, and of Bichat, may command our reverence for their successful interrogation of nature; but those of Sydenham, of Pott, and of Astley Cooper, have an equal title to our respect as benefactors of mankind. To these latter posterity will add the name of Mr. Brodie, recognizing in his works the vivid descriptions of Sydenham, the sound judgment of Pott, and the extensive experience of Cooper.

